# Effects of Chitosan on Drug Load and Release for Cisplatin–Hydroxyapatite–Gelatin Composite Microspheres

**DOI:** 10.3390/polym17111485

**Published:** 2025-05-27

**Authors:** Meng-Ying Wu, I-Fang Kao, Shiow-Kang Yen

**Affiliations:** 1 Department of Materials Science and Engineering, National Chung Hsing University, Taichung 402, Taiwan; slimu16882013@gmail.com (M.-Y.W.);; 2 Department of Orthopedics, Taichung Armed Forces General Hospital, Taichung 404, Taiwan; 3 Department of Gerontological Health Care, Central Taiwan University of Science and Technology, Taichung 406, Taiwan; 4 Department of Natural Biotechnology, Nanhua University, Chiayi 622, Taiwan; 5 Department of Dental Technology and Material Science, Central Taiwan University of Science and Technology, Taichung 406, Taiwan

**Keywords:** hydroxyapatite–gelatin microspheres, cone-like pores, cisplatin, drug entrapment efficiency, chitosan, release duration

## Abstract

Cisplatin, a widely used chemotherapeutic agent, is limited by its poor bioavailability, rapid systemic clearance, and severe side effects. To overcome these limitations, hydroxyapatite–gelatin composite microspheres were developed to improve drug entrapment efficiency (DEE) and provide sustained drug release. Various formulations were prepared by incorporating chitosan either by mixing once or through a sequential coating strategy. By adjusting the loading procedure, the DEE increased from 58% to 99%. The composite microsphere effectively controlled the total drug release duration, extending it from one month to over 5 months. Moreover, the MTT assay demonstrated that all samples effectively inhibited cell growth, with cell viability reduced to less than 20% after 2 weeks of experimentation. These findings demonstrate that the sequential chitosan coating method offers superior drug entrapment and prolonged release compared to mixing chitosan once, exhibiting its potential as a sustained drug delivery system for cancer treatment.

## 1. Introduction

Cancer remains a leading cause of death globally, with approximately 10 million lives lost in 2020 alone, and the incidence is expected to rise steadily over the coming decades [1]. Treatment options such as surgery, radiotherapy, and chemotherapy are widely used, but each comes with notable limitations [2,3]. Among them, chemotherapy continues to be a cornerstone of cancer treatment despite significant challenges related to toxicity, multidrug resistance, and poor drug specificity [4,5,6,7].

Cisplatin (CDDP), a platinum-based chemotherapeutic agent, has been widely used in the treatment of various cancers, including ovarian, testicular, bladder, and bone cancers [3]. Its mechanism involves crosslinking DNA, thereby disrupting DNA replication and inducing apoptosis in cancer cells [8,9]. Since its Food and Drug Administration (FDA) approval in 1978, CDDP has remained one of the most effective chemotherapeutic drugs [10]. However, its clinical application is limited due to significant side effects such as nephrotoxicity, hepatotoxicity, ototoxicity, and gastrointestinal disorders [11,12]. Additionally, CDDP has low lipophilicity and reduced bioavailability, which further complicates effective administration [13].

To overcome these limitations, the development of drug delivery systems (DDSs) has become an area of intense research [6,14,15,16]. Nanoparticle-based DDSs offer promising solutions by improving drug loading, stability, and controlled release while minimizing off-target toxicity [10,17]. These systems often utilize materials such as liposomes, polymeric nanoparticles, and inorganic carriers to encapsulate and deliver chemotherapeutic agents [4,18,19].

Among the various inorganic materials, hydroxyapatite (HAp) has emerged as a promising drug carrier due to its biocompatibility, bioactivity, and porous structure, making it suitable for drug encapsulation and sustained release [20]. HAp is the main inorganic component of human bones and teeth, and its surface chemistry allows for hydrogen bonding and electrostatic interaction with drug molecules [21,22]. It is also less cytotoxic compared to other inorganic nanocarriers and supports targeted drug delivery, especially in bone cancer treatment [23,24].

Chitosan, a natural biopolymer, has also gained attention in drug delivery for its biodegradability and strong hydrogen bonding capacity [25,26]. It can enhance drug entrapment, modulate release profiles, and improve biocompatibility of DDS systems [27,28,29,30]. The combination of HAp and chitosan offers complementary advantages: while HAp provides structural and adsorption capacity, chitosan contributes to improved drug retention and prolonged release [31].

Given the clinical limitations of cisplatin, including its systemic toxicity and rapid clearance, DDSs can enhance its therapeutic efficacy while minimizing adverse effects. While HAp offers favorable properties such as biocompatibility and drug adsorption capacity, its drug retention ability remains suboptimal. On the other hand, chitosan provides strong hydrogen bonding and biodegradability, making it a valuable polymeric component in DDS design.

Previous studies have demonstrated the successful encapsulation of both hydrophobic paclitaxel (PTX) [31] and hydrophilic doxorubicin (DOX) [32] using HAp–gelatin microspheres. Through chitosan incorporation or adjustment of the drug-loading sequence, drug entrapment efficiencies (DEE) exceeding 90% and prolonged release durations up to several weeks or even over 6 months have been achieved. However, although both DOX and CDDP are hydrophilic, CDDP may behave differently in the same carrier system. Therefore, this study aims to identify an optimal formulation for CDDP with high entrapment efficiency and sustained release. The findings could offer new insights into the rational design of HAp-based drug delivery systems for improved chemotherapeutic performance.

## 2. Materials and Methods

### 2.1. Hydroxyapatite Microspheres (HAp)

#### 2.1.1. Synthesis of HAp

The synthesis of hydroxyapatite (HAp) microspheres was performed according to a previously established protocol [31,32,33,34]. Briefly, a 0.025 M aqueous solution of ammonium dihydrogen phosphate ((NH_4_)H_2_PO_4_, SHOWA, Tokyo, Japan) was mixed with a 0.042 M aqueous solution of calcium nitrate tetrahydrate (Ca(NO_3_)_2_·4H_2_O, SHOWA, Tokyo, Japan), along with a 4 wt.% gelatin solution (Sigma-Aldrich, St. Louis, MO, USA). The mixture was stirred thoroughly until homogeneous and then allowed to stand undisturbed. Upon precipitation of the gelatin-hydroxyapatite complex, the precipitate was separated by filtration, dried in an oven, and stored at room temperature for further use.

#### 2.1.2. Characterization of Synthesized HAp

To analyze the surface morphology of the synthesized HAp microspheres, both before and after drug loading, scanning electron microscopy (SEM, JSM-5400, JEOL, Tokyo, Japan) was employed. The samples—including pure HAp microspheres and CDDP-loaded composites—were mounted onto SEM stubs using conductive carbon tape. No gold coating was applied prior to imaging, except for one chitosan-based sample without HAp, which required gold sputtering to improve conductivity. All SEM observations were performed under low voltage (10 kV) to minimize charging artifacts, and images were collected to evaluate particle size and surface morphology.

### 2.2. Drug-Loading Protocols

Different drug-loading methods were employed to incorporate cisplatin (CDDP, MedChemExpress, Monmouth Junction, NJ, USA) into the HAp microspheres.

C1–C5: C1, C2, C3, C4, and C5 were each prepared by directly mixing 1.5 mg of CDDP in aqueous chitosan (Sigma-Aldrich, St. Louis, MO, USA, MW 50~100 kDa, deacetylation degree ≥ 75%) solutions dissolved in 0.33 vol% acetic acid, with increasing concentrations of 0%, 0.125%, 0.25%, 0.5%, and 1%, respectively, along with 15 mg of HAp, followed by lyophilization.

C6: First, 1.5 mg CDDP was loaded into 15 mg HAp and lyophilized, same as C1. Subsequently, the CDDP-HAp powder was suspended in 1% chitosan, followed by another freeze-drying step.

C7: Prepared by mixing 1.5 mg of CDDP with 1% chitosan solution, without the addition of HAp. This group served to verify the drug-loading capability of chitosan alone. Due to its low conductivity and absence of HAp, this sample was gold-coated prior to SEM imaging.

C8 and C9: Followed the same procedure as C6, but with increased initial drug concentrations. C8 contained 3 mg of CDDP, and C9 contained 5 mg of CDDP, both with 15 mg of HAp and 1% chitosan.

**Figure 1:**
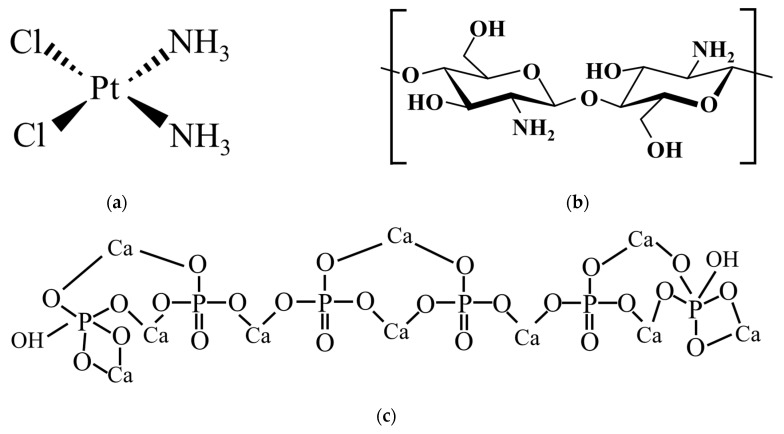
Chemical structures of (**a**) CDDP, (**b**) chitosan, and (**c**) HAp.

All mixing and incubation steps were performed at 37 °C in a water bath with continuous agitation at 80 rpm. After lyophilization, the samples were collected and stored at −20 °C until further analysis. The representative chemical structures of CDDP, chitosan, and HAp are shown in Figure 1.

### 2.3. Drug-Loading Content (DLC) and Drug Encapsulation Efficiency (DEE) Analysis

#### 2.3.1. UV-Visible Calibration Curve Establishment for CDDP Quantification

To determine the concentration of CDDP in the experimental samples, a calibration curve was established. Standard solutions of CDDP ranging from 0, 3.125, 6.25, 12.5, 25, 50, 75, 100, and 150 ppm were prepared in deionized water. The absorbance of each solution was measured using a UV-visible spectrophotometer at 207 nm, which corresponds to the absorption wavelength of CDDP. The obtained absorbance values were plotted against the known concentrations to generate a standard calibration curve.

#### 2.3.2. Assessment of Cisplatin Loading and Entrapment Performance

For drug-loading assessment, the concentration of unbound CDDP in the supernatant was determined using UV-visible spectroscopy, and the values were applied to the calibration curve to calculate the drug-loading content (DLC) and drug encapsulation efficiency (DEE) using the formulas below:

DEE (%) = (Initial CDDP amount − CDDP amount in the supernatant) × 100/initial CDDP amount,(1)

DLC (%) = (Initial CDDP amount − CDDP amount in the supernatant) × 100/carrier amount,(2)

### 2.4. In Vitro Drug Release Study

To evaluate drug release kinetics, 15 mg of each sample was placed into a 15 mL centrifuge tube containing 10 mL of phosphate-buffered saline (PBS, Gibco, MA, USA). The tubes were incubated in a water bath at 37 °C with continuous shaking at 80 rpm. At each time point, 1 mL of the supernatant was withdrawn for analysis, and an equal volume of fresh PBS was added. The concentration of released CDDP was determined using UV-visible spectroscopy.

### 2.5. Cell Cytotoxicity Assay

The cytotoxic effects of the drug-loaded HAp microspheres were assessed using the G292 cell line. Cells were cultured in McCoy’s 5A (modified, Gibco, Waltham, MA, USA) medium supplemented with 10% fetal bovine serum (Biological Industries, Beit Haemek, Israel) under a 5% CO_2_ atmosphere at 37 °C.

MTT (3-[4,5-dimethylthiazol-2-yl]-2,5 diphenyl tetrazolium bromide) assays were performed according to ISO 10993-5 guidelines [35] to evaluate cell viability. Each drug-loaded sample, as well as free CDDP powder (control group), was co-cultured with 10^4^ cells/100 μL of G292 cells in a 24-well plate, with a fixed drug concentration of 5 ppm. Cells were incubated for 1, 4, 7, and 14 days. At each time point, the culture medium was removed, and an equal volume of MTT solution (GoldBio, St. Louis, MO, USA) was added to each well, followed by a 3-h incubation at 37 °C. The MTT solution was then replaced with an equal volume of DMSO (Panreac Applichem, Barcelona, Spain) to dissolve the formazan crystals formed by viable cells. Absorbance was measured using an ELISA reader (Stat Fax-2100, Awareness Technology, Inc., Palm City, FL, USA), and results were compared to the control group. All experiments were performed in triplicate.

## 3. Results and Discussion

### 3.1. Surface Morphology of HAp

**Figure 2:**
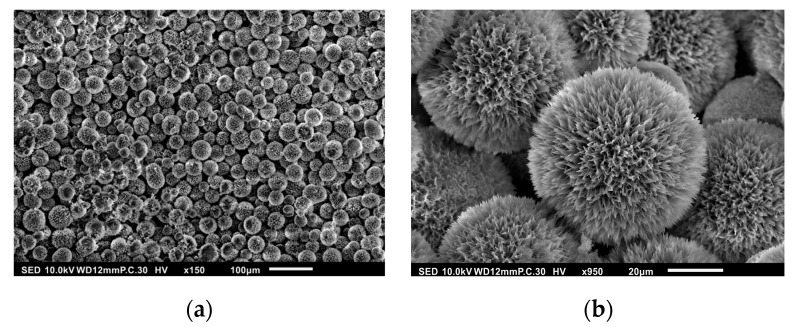
SEM images of synthesized HAp composite microspheres at (**a**) 150× and (**b**) 950× magnifications.

SEM images of the synthesized HAp microspheres demonstrate the successful formation of well-defined spherical structures. The low-magnification image as seen in Figure 2a reveals a high yield of microspheres with relatively uniform size distribution. At higher magnification, as shown in Figure 2b, a porous and rough surface morphology is observed, which is characteristic of HAp synthesized via the gelatin-assisted precipitation method. The rough and porous nature of the microspheres is beneficial for drug loading, as it provides a higher surface area for drug adsorption [32]. The presence of hierarchical nanostructures on the microsphere surface suggests a potential increase in drug encapsulation efficiency. Additionally, the uniformity of the microspheres indicates a well-controlled synthesis process, which is essential for reproducibility in biomedical applications.

The observed morphology aligns with previous reports on gelatin-mediated HAp synthesis, confirming that the use of gelatin as a templating agent effectively facilitates the self-assembly of HAp into micro-spherical structures [31,32].

### 3.2. Drug Entrapment Efficiency (DEE) and Drug-Loading Content (DLC)

#### 3.2.1. Calibration Curve for CDDP Analysis

The absorbance values obtained from UV-visible measurements of 0–150 ppm CDDP solutions were used to construct a calibration curve with the equation y (Absorbance Units) = 0.0162 × (ppm) + 0.025 and a coefficient of determination R^2^ of 0.9952. This curve was subsequently used to determine unknown CDDP concentrations in the supernatant of drug-loading and release experiments.

#### 3.2.2. DLC and DEE Analysis

**Figure 3:**
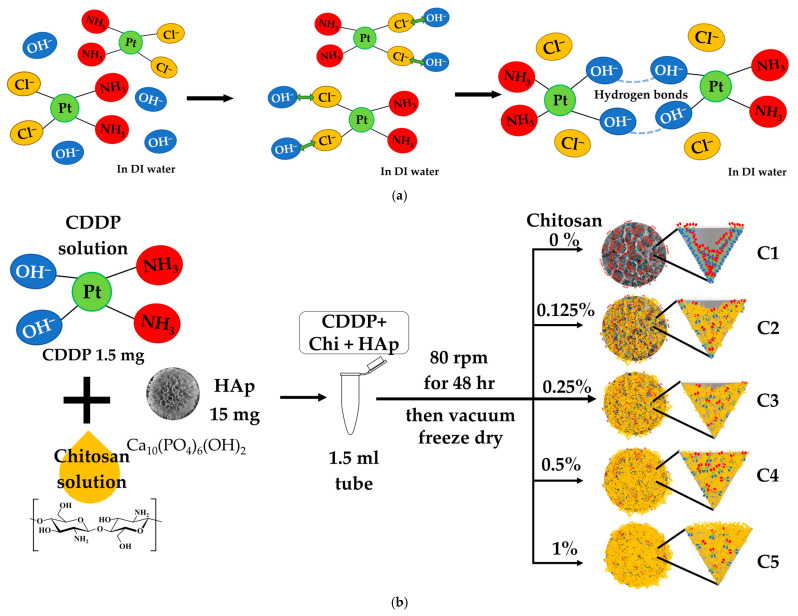

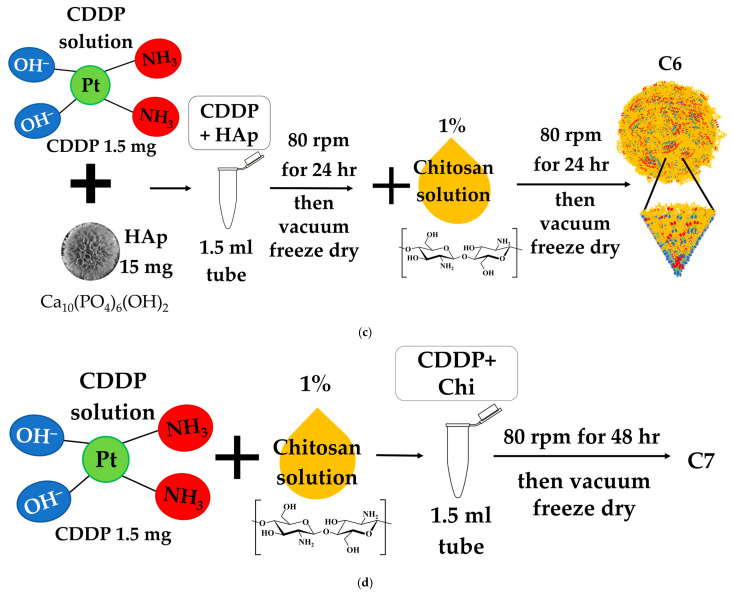
Illustration of (**a**) CDDP dissolution in deionized water; and detailed preparation procedures for (**b**) C1, C2, C3, C4, and C5, (**c**) C6, and (**d**) C7.

Figure 3a shows when cisplatin (cis-[PtCl_2_(NH_3_)_2_]) undergoes hydrolysis after dissolving in water, in which one or both chloride ions (Cl^−^) are replaced by hydroxide ions (OH^−^), forming cis-[Pt(OH)_2_(NH_3_)_2_] [36]. This structural transformation results in the partial or complete conversion of Cl^−^-containing CDDP into its OH^−^-substituted form, making it easier for CDDP molecules to form stable hydrogen bonds either with each other or with other materials such as chitosan or HAp. In addition, this reaction releases protons (H^+^), which explains why CDDP solutions typically exhibit weak acidity (pH 5.54). The detailed procedures for deriving samples C1, C2, C3, C4, and C5 are shown in Figure 3b; samples C6 and C7 are depicted in Figure 3c and Figure 3d, respectively. The DEE and DLC are listed in Table 1.

The DEE values indicate a clear trend influenced by the presence and concentration of chitosan. The HAp microspheres without chitosan (C1) exhibited a DEE with a minimal standard deviation of 58.33% ± 0.88, suggesting a moderate drug entrapment capability. When chitosan at various concentrations was mixed directly with CDDP and HAp during the loading process, a decreasing trend in DEE was observed. Specifically, C2 had a DEE of 50.24% ± 1.47, C3 had 48.36% ± 1.47, C4 had 40.17% ± 1.11, and C5 showed the lowest DEE at 32.91% ± 2.05. By only tuning the process of loading into the sequential chitosan coating method (C6), the result showed the highest DEE of 99.43% ± 0.21, indicating highly efficient drug encapsulation. C7 was specifically prepared to confirm chitosan’s ability to capture CDDP independently. The rationale behind C6 was that after lyophilization of C1, the subsequent addition of 1% chitosan allowed for the adsorption of any unencapsulated CDDP onto the chitosan-coated HAp, leading to the observed high DEE. To test this, C7 was prepared using chitosan and CDDP without HAp. The results showed that C7 exhibited a high DEE of 75.07%, supporting the hypothesis that chitosan alone can effectively capture and retain CDDP. To verify the potential for increasing DLC rather than being limited to approximately 9.94%, as observed in C6, additional formulations C8 and C9 were tested. These samples followed the same procedure as C6 but with increased initial drug concentrations: C8 contained 3 mg of CDDP, and C9 contained 5 mg of CDDP. The goal was to determine whether increasing the drug amount would enhance DLC while maintaining high DEE. Results showed that increasing the initial drug amount significantly raised DLC values, with C8 reaching 18.51% and C9 reaching 29.97%.

The interaction between CDDP and HAp is primarily mediated by hydrogen bonds. However, due to the limited amount of OH^−^ groups on the HAp surface, its drug-loading capacity for CDDP is somewhat restricted. In C1, HAp was able to absorb a portion of CDDP but could not fully load all the drug, as shown in Figure 3b. C7 showed that chitosan alone could also bind CDDP through hydrogen bonds, suggesting a relatively strong binding capacity for CDDP. However, without the structural support of HAp, the chitosan matrix may be short of the stability required for sustained drug release, as depicted in Figure 3d. For C2–C5, the decreasing trend in DEE may be attributed to the initial binding of chitosan to HAp, partially filling the cone-like pores of HAp and limiting its capacity to absorb CDDP. The OH^−^ groups in chitosan may also compete with HAp for hydrogen bonds with CDDP, reducing the overall drug-loading efficiency, as indicated in Figure 3b. C6 exhibited superior performance, likely due to its loading sequence—HAp first absorbs CDDP and is then coated with chitosan. The added chitosan can capture residual CDDP not initially absorbed by HAp, thereby maximizing entrapment efficiency, as illustrated in Figure 3c. As demonstrated by C7, 1% chitosan was able to absorb approximately 75% of 1.5 mg CDDP, suggesting that further increases in drug concentration alone may not be sufficient to improve DEE or DLC in samples like C8 and C9. Instead, increasing the chitosan concentration may be necessary to enhance drug entrapment efficiency and drug-loading content at higher drug doses.

The drug entrapment and loading efficiency of CDDP were significantly influenced by the presence, concentration, and incorporation method of chitosan. Directly mixing chitosan during the loading process (C2–C5) resulted in reduced DEE, whereas the sequential chitosan coating approach (C6) achieved the highest DEE by capturing residual drug. Chitosan alone (C7) demonstrated good binding capacity, and increasing drug concentration (C8, C9) successfully improved the DLC.

**Figure 4:**
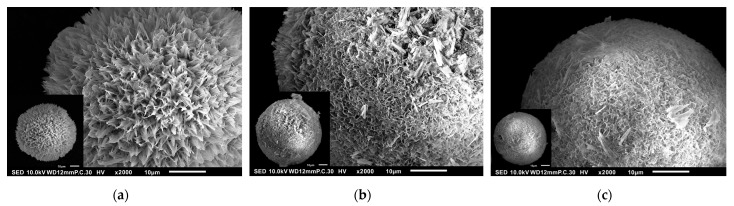

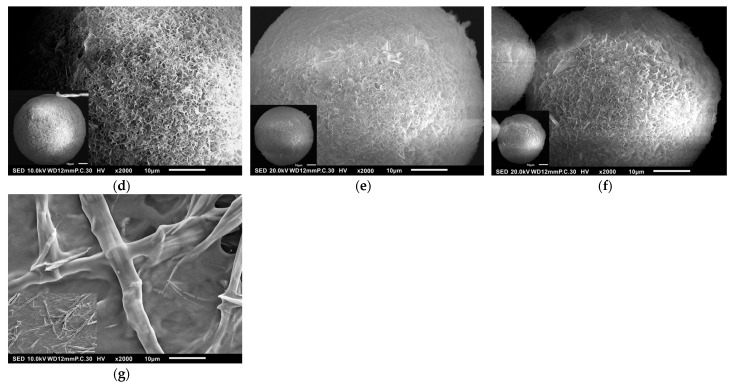
SEM observation of samples (**a**) C1, (**b**) C2, (**c**) C3, (**d**) C4, (**e**) C5, (**f**) C6, and (**g**) C7.

The SEM images of C1, C2, C3, C4, C5, C6, and C7 are shown in Figure 4a–g. The morphology of C1 to C6 generally corresponds to the simulated model shown in Figure 3. Cone-like pores are distinctly visible in sample C1, while other samples show fewer, smaller, and shallower pores in comparison. As the amount of chitosan increases, the cone-like pores become progressively less visible, suggesting that chitosan may partially fill or obscure the pore structures. C7, comprising only CDDP and chitosan without HAp, exhibits irregular, flake-like, or networked aggregates rather than spherical microspheres. This aggregated morphology is likely due to hydrogen bonding and potential coordination interactions between CDDP and the amino and hydroxyl groups of chitosan.

### 3.3. CDDP Release Profiles

**Figure 5:**
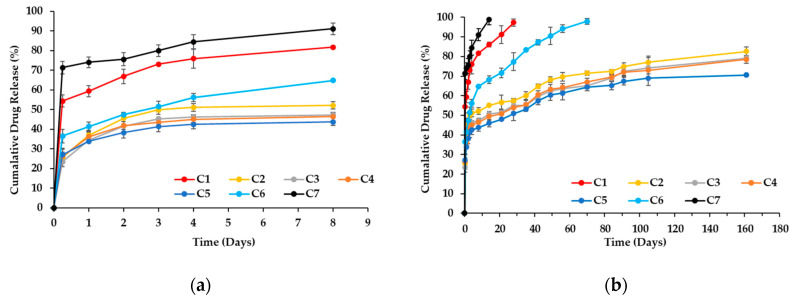
Cumulative drug release profiles in (**a**) 8 days, and (**b**) full release curves up to Day 161.

Drug release studies were conducted over a period of up to 5 months to evaluate the sustained release characteristics of the different formulations. The cumulative drug release (CDR) values demonstrated distinct release behaviors depending on chitosan concentration and loading method, as shown in Figure 5.

C1 (the red one) exhibited the highest initial burst release, with 59.36% of CDDP released within the first 24 h, increasing to 97.34% by Day 28. In contrast, C6 (the light blue one) showed a more controlled release, with 41.29% released at 24 h, followed by a gradual increase to 97.9% at 70 days. C2 (the yellow one) released 36.9% of CDDP in 24 h and 82.65% by Day 161. C3 (the gray one), C4 (the orange one), and C5 (the dark blue one) exhibited further delayed release profiles, with C5 achieving 70.56% cumulative release by Day 161. A trend of slower release was observed as chitosan concentration increased. C7 (the black one) exhibited a distinct release behavior, with 58.57% of the drug released within the first 1 h, increasing to 74.01% by Day 1 and reaching 98.99% by Day 14.

The release profile of group C1 indicates that the lower number of OH^−^ groups on HAp, compared to chitosan as shown in Figure 1, results in weaker drug binding, leading to easier release of CDDP and a faster release rate. C7, lacking the structural protection of HAp’s cone-like pores, exhibited a higher initial burst compared to C1. Without the shielding effect of HAp, the CDDP/chitosan complex in C7 was fully exposed to the release solution, leading to the fastest release, with complete drug release occurring within 2 weeks. In contrast, C1, with the protective structure of HAp cones, extended the release duration to one month. Both C6 and C2–C5 involved chitosan in the drug-loading process, but C6 consistently exhibited a faster release rate than C2–C5. Since the chitosan introduced in the second step of C6 formed fewer hydrogen bonds with HAp, as CDDP had already occupied some OH^−^ groups of HAp, the subsequent chitosan/CDDP complexes dissolved more readily, resulting in a faster release than that observed in C2–C5. C2–C5 showed relatively slower release rates. This may be attributed to the more hydrogen bonds between chitosan and HAp formed during the initial preparation process. The cone-like pores of HAp further affected the drug diffusion, allowing CDDP to be more stably retained and thus lowering the release rate.

Compared to other studies utilizing carriers for CDDP loading and releasing, HAp-based drug carrier systems developed in this study demonstrate notable advantages as listed in Table 2. In terms of the DLC, this study achieved a value of 29.97%, the highest among the reviewed works. In contrast, Wang et al., using folic acid-modified nanoparticles (FA-NP), reported DLC values of only 11.25% [37], while Xiang et al. reported even the lower value of 7.72% with PEG-GA core-shell nanoparticles [38]. Moghadam et al., employing mesoporous silica nanoparticles, achieved a DLC of 20.7% [11]. Although Xiang et al.’s study showed a high DEE of 99.2%, its low DLC suggests limited actual drug-carrying capacity [38].

In terms of drug release, Wang et al.’s system released 75% of CDDP within 36 h [37], while Xiang et al. and Moghadam et al. exhibited little drug release beyond 20 and 50 h, respectively [11,38]. In contrast, our system demonstrates well-controlled release behavior, extending from 20 days up to 161 days, which offers superior sustainability and stability—especially suitable for the prolonged treatment of cancer. Taken together, our system exhibits superior performance in the DLC and controllable release kinetics, making it a highly promising platform for the long-term and effective delivery of CDDP in cancer therapy.

Our previous studies demonstrate there is research about loading cancer drugs such as doxorubicin (DOX) [32] and paclitaxel (PTX) [31] onto HAp. Although both DOX and CDDP are hydrophilic chemotherapeutics, their interactions with HAp and chitosan differ significantly. Specifically, mixing CDDP directly with chitosan—following the same method previously used for DOX—resulted in the reduced DEE as the chitosan concentration increased, as listed in Table 1 C1, C2, C3, C4, and C5. In contrast, the application of a sequential chitosan coating strategy—similar to that used for PTX—significantly enhanced CDDP loading, raising the DEE from approximately 58% to 99.43% such as C6. In terms of drug release duration, unmodified CDDP formulations completed release within one month, compared to only 7 h for DOX. However, when chitosan was incorporated, both drugs exhibited a release duration longer than 23 weeks, while PTX maintained long-term release regardless of chitosan addition. These results highlight that even within the same carrier system, different drugs exhibit distinct release behaviors due to the varying interactions with chitosan and HAp. Therefore, strategic optimization—such as adjusting chitosan concentration, loading sequence, or incorporating multistep processing—is essential for maximizing the therapeutic potential of CDDP-loaded HAp composite systems.

### 3.4. Cell Viability Through MTT Assay

**Figure 6:**
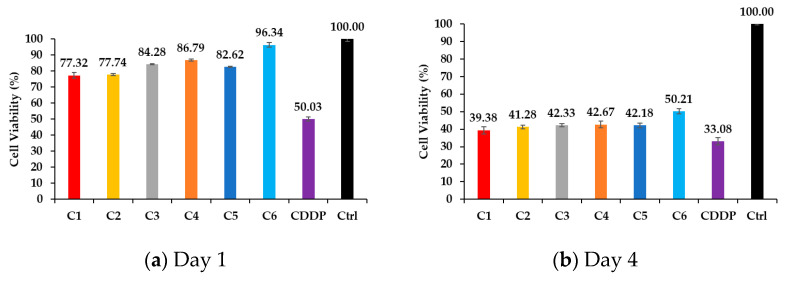

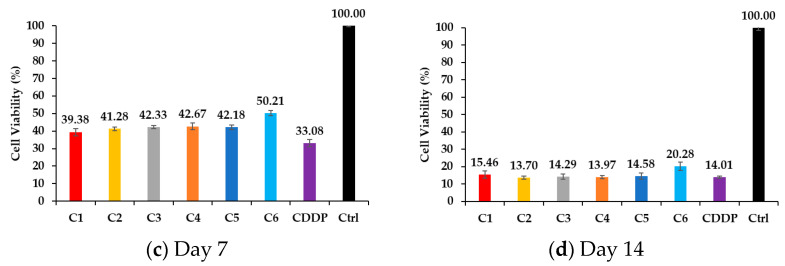
Cell viability assessed using MTT assay on (**a**) Day 1, (**b**) Day 4, (**c**) Day 7, and (**d**) Day 14.

The MTT assay results indicate that all drug-loaded samples exhibited a time-dependent decrease in cell viability as seen in Figure 6, reflecting the sustained release and cytotoxic effects of CDDP. On Day 1, C6 (the light blue one) showed the highest cell viability at 96.34%, while the group treated with free CDDP showed a viability of 50.03%, indicating immediate cytotoxicity. C1 (the red one), C2 (the yellow one), C3 (the gray one), C4 (the orange one), and C5 (the dark blue one) displayed intermediate values ranging from 77.31% to 86.79%, suggesting a delayed but effective cytotoxic response compared to C1, which showed an earlier yet less intense initial effect. By Day 4, a significant reduction in cell viability was observed across all groups. C6 and C1 showed evident cytotoxicity, with viabilities decreasing to 50.20% and 39.38%, respectively. The free CDDP group exhibited the strongest early effect, with viability dropping to 33.08%. Meanwhile, C2 to C5 showed a similar range of viability between 41.27% and 42.66%, indicating a gradual increase in cytotoxicity likely due to their sustained drug release profiles. On Day 7, viability further decreased across all samples. C1 and free CDDP both exhibited similar levels of cytotoxicity, with viabilities at 29.18% and 28.25%, respectively. C6 maintained slightly higher viability at 32.99%, confirming its continued controlled drug release. C2 to C5 followed a comparable trend, with values ranging from 26.89% to 28.18%, collectively demonstrating a steady cytotoxic effect. By Day 14, cell viability reached its lowest levels. C1 and free CDDP showed comparable cytotoxicity, with viabilities at 15.46% and 14.01%, respectively. This suggests that free CDDP exerted its full effect early, while C1 achieved similar cytotoxicity more gradually. C6 retained slightly higher viability at 20.27%, supporting the idea of delayed but effective long-term drug release. C2 to C5 ended with viabilities between 13.69% and 14.58%, indicating a consistent and controlled cytotoxic effect over time.

Due to C6 exhibiting the highest DEE among all groups, a smaller quantity of the sample was needed to provide the same fixed drug dose used across all MTT wells. As a result, the solid surface area covered by the C6 sample was smaller, exposing a greater portion of the polystyrene well bottom. This additional exposure likely facilitated cell adhesion and may help explain the notably higher cell viability observed for C6 on Day 1. In summary, C6 exhibited the most controlled and sustained release profile, leading to delayed cytotoxic effects compared to free CDDP. The C2–C5 samples collectively demonstrated gradual drug release, ensuring a steady decline in cell viability without an abrupt burst effect. C1 closely mirrored the effect of free CDDP but with a delayed onset of cytotoxicity. These results highlight the role of chitosan and HAp in modulating drug release kinetics and cytotoxic effects over time, reinforcing their potential as a sustained drug delivery system for cancer treatment.

## 4. Conclusions

This study successfully developed and characterized various formulations of cisplatin (CDDP)-loaded hydroxyapatite–gelatin composite microspheres to evaluate their potential as a sustained drug delivery system. The results demonstrated that the entrapment efficiency and release profiles were significantly influenced by the presence, concentration, and incorporation method of chitosan.

Among all formulations, C6, prepared using a sequential chitosan coating method, exhibited the highest drug entrapment efficiency (DEE) of 99.43% and a drug-loading content (DLC) of 9.94%. Directly incorporating chitosan once during the loading process (C1–C5) resulted in a decreasing trend in DEE due to initial binding of chitosan to HAp, limiting further drug adsorption. C7, which was prepared using only chitosan and CDDP without HAp, confirmed that chitosan alone can effectively bind CDDP, achieving a DEE of 75.07%, though lacking the structural support provided by HAp. Higher drug concentrations in C8 and C9 increased the DLC to 18.51% and 29.97%, respectively. However, further improvement in DEE would require tuning the chitosan concentration.

The duration of drug release is influenced by the bonding interactions among chitosan, HAp, and CDDP, as well as the degradation rate of chitosan. C7 released its drug within approximately 2 weeks due to the maximum contact area between the Chitosan/CDDP complex and the release solution, leading to rapid drug release. C1 extended the release time to one month because of the hydrogen bonding between HAp and CDDP, along with the protective effect provided by the cone-like pores of HAp. C6 exhibited a prolonged release of about two and a half months due to the protective role of the HAp cone-like pores and the formation of hydrogen bonds between the chitosan layer and HAp, both of which slowed down the degradation rate of chitosan compared to C7. C2–C5 exhibited the longest release duration, extending up to approximately 5 months. This is attributed to the formation of more hydrogen bonds between chitosan and HAp, slowing down the degradation of chitosan.

The MTT assay confirmed that the cytotoxicity of CDDP was not altered by different loading procedures. All samples effectively inhibited cell growth over time, demonstrating consistent drug efficacy regardless of loading procedures.

These findings indicate the importance of formulation design and material interactions in optimizing drug-loading and delivery performance. The sequential chitosan coating strategy proved effective in enhancing CDDP entrapment and modulating release kinetics, making C6 a promising candidate for sustained drug delivery applications.

## Figures and Tables

**Table 1:** Drug entrapment efficiency (DEE) and drug-loading content (DLC) of C1, C2, C3, C4, C5, C6, C7, C8, and C9.

	DEE (%)	DLC (%)
C1	58.33 ± 0.88	5.83 ± 0.09
C2	50.24 ± 1.47	5.02 ± 0.15
C3	48.36 ± 1.47	4.84 ± 0.15
C4	40.17 ± 1.11	4.02 ± 0.11
C5	32.91 ± 2.05	3.29 ± 0.21
C6	99.43 ± 0.21	9.94 ± 0.02
C7	75.07 ± 3.57	34.17 ± 7.89
C8	84.63 ± 0.69	18.51 ± 0.15
C9	74.34 ± 0.99	29.97 ± 0.4

**Table 2:** Comparison of some CDDP-loaded drug delivery systems.

	[37]	[38]	[11]	This Study
Carrier	FA-NP	PEG-GA2	Silica nanoparticles	Hydroxyapatite
Particle size	133.4 nm	135 ± 8.7 nm	100–200 nm	40–50 µm
DLC (%)	11.25 ± 0.35	7.72	20.7	29.97
DEE (%)	69.5 ± 1.09	99.2	17	74.34

## Data Availability

Data are contained within the article.
